# Microbial community composition of deep-sea corals from the Red Sea provides insight into functional adaption to a unique environment

**DOI:** 10.1038/srep44714

**Published:** 2017-03-17

**Authors:** Till Röthig, Lauren K. Yum, Stephan G. Kremb, Anna Roik, Christian R. Voolstra

**Affiliations:** 1 Red Sea Research Center, Division of Biological and Environmental Science and Engineering (BESE), King Abdullah University of Science and Technology (KAUST), Thuwal 23955-6900, Saudi Arabia

## Abstract

Microbes associated with deep-sea corals remain poorly studied. The lack of symbiotic algae suggests that associated microbes may play a fundamental role in maintaining a viable coral host via acquisition and recycling of nutrients. Here we employed 16 S rRNA gene sequencing to study bacterial communities of three deep-sea scleractinian corals from the Red Sea, *Dendrophyllia sp., Eguchipsammia fistula*, and *Rhizotrochus typus*. We found diverse, species-specific microbiomes, distinct from the surrounding seawater. Microbiomes were comprised of few abundant bacteria, which constituted the majority of sequences (up to 58% depending on the coral species). In addition, we found a high diversity of rare bacteria (taxa at <1% abundance comprised >90% of all bacteria). Interestingly, we identified anaerobic bacteria, potentially providing metabolic functions at low oxygen conditions, as well as bacteria harboring the potential to degrade crude oil components. Considering the presence of oil and gas fields in the Red Sea, these bacteria may unlock this carbon source for the coral host. In conclusion, the prevailing environmental conditions of the deep Red Sea (>20 °C, <2 mg oxygen L^−1^) may require distinct functional adaptations, and our data suggest that bacterial communities may contribute to coral functioning in this challenging environment.

A growing number of studies support the notion that bacteria associated with multicellular hosts provide important functions related to metabolism, immunity, and environmental adaptation (among others)^1^. This might be of particular significance in ‘extreme’ environments, such as the deep sea. Nevertheless, only few studies investigated the diversity and function of bacteria associated with deep-sea corals. Similar to shallow corals^2^, deep-sea coral mucus stimulates microbial activity in the immediate vicinity of the coral host, suggestive of nutrient cycling via a microbial loop and thereby increasing ecosystem productivity^3^. However, deep-sea coral ecology is fundamentally different from the ecology of shallow-water corals. Deep-sea corals lack light, are exposed to elevated pressures, and usually reside at cold(er) temperatures^4^. Most importantly, they do not associate with photosynthetic algal symbionts in the genus *Symbiodinium*, and thereby lack a carbon source that provides the majority of the energetic requirements of shallow-water corals. Consequently, associated microbial communities of deep-sea corals are thought to play an essential role for host metabolism, e.g. by supplementing host feeding via fixing and recycling of nitrogen and carbon^5^,^6^,^7^. In support of this, in shallow-water corals increased bacterial diversity with increasing water depth has been suggested as a mechanism to aid nutrient acquisition^8^.

Studies of Red Sea corals, along with those in the Persian/Arabian Gulf, are of interest in light of global climate change because corals in this region thrive at summer temperatures (>31 °C) that are hostile for corals from other regions^9^,^10^. The tolerance to high temperature extends down to the depths of the Red Sea, where deep-sea corals are found at >20 °C^11^. The discovery of deep-sea corals at this temperature prompted a reconsideration of environmental limitations of deep-sea corals, previously considered to be limited to temperatures of 4–14 °C^4^,^12^. Besides these unusual high temperatures, a study from the central Red Sea by Roder *et al*.^11^ reported higher salinity (40.5 PSU) and lower dissolved oxygen (1–2 mg L^−1^) levels, compared to other deep-sea coral environments.

To date, only few studies investigated microbial communities associated with deep-sea reef communities. At present, studies have focused on octocorals^7^,^13^,^14^,^15^ and two cold-water scleractinian corals, *Lophelia pertusa* and *Madrepora oculata*^5^,^16^,^17^,^18^,^19^,^20^,^21^,^22^,^23^,^24^,^25^. These studies support the presence of species-specific microbiomes^7^,^14^,^23^, but at the same time identified highly variable bacterial community structures depending on phenotype, spatial and temporal scales, and even on individual polyps within a colony^5^,^18^,^19^,^21^,^23^.

To gain insight into bacterial community structure associated with deep-sea scleractinian corals from the Red Sea, we conducted a 16S rRNA gene survey on specimens from *E. fistula, Dendrophyllia* sp., and *R. typus*. We were interested to elucidate how bacterial association differs between coral hosts, and also, whether we find support for a bacterial contribution to functional adaptation to the distinct environment of the Red Sea.

## Results

### Microbial community composition of deep-sea corals and surrounding seawater

**Figure 1:**
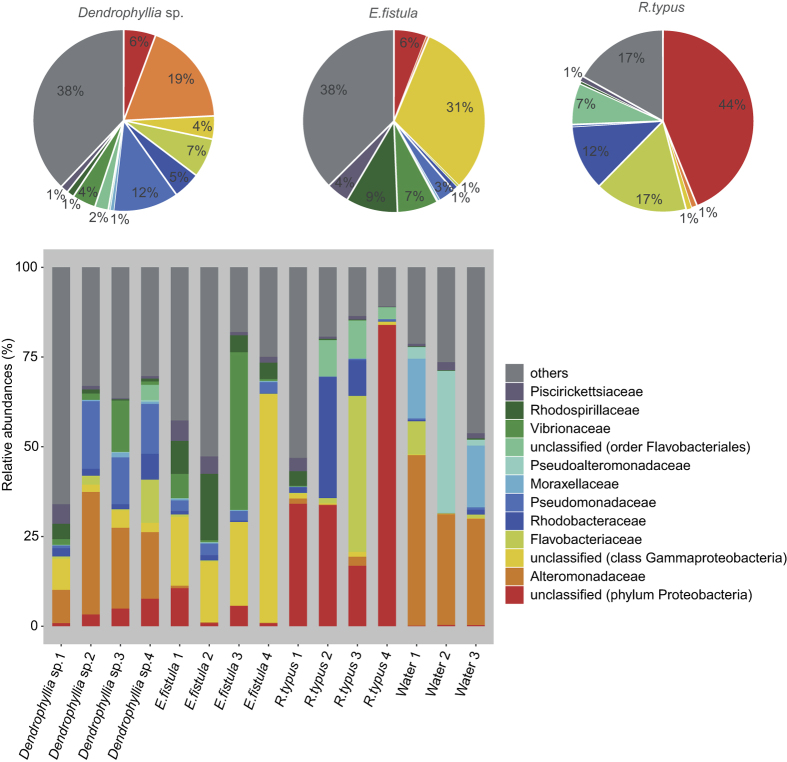
Bacterial community composition of deep-sea corals and seawater from the Red Sea on the bacterial family level (Greengenes database, bootstrap ≥60). Pie charts denote average bacterial abundance across replicates for the three coral species. The taxonomy bar plot denotes relative bacterial abundance across species replicates and seawater. Each color represents one of the 12 most abundant families (overall sequence count) in all samples. All other taxa are grouped under category ‘others’.

A total of 852,313 sequences were processed and, after filtering, yielded 554,088 sequences across 15 samples (i.e., 4 samples of *Dendrophyllia* sp., 4 samples of *E. fistula*, 4 samples of *R. typus*, and 1 water sample for each of the 3 collection sites). To evaluate bacterial community composition across samples, we classified all sequences to the family level (Fig. 1). The bacterial community composition of coral samples was markedly different from seawater samples that appeared similar across sampling sites (Fig. 1). Coral community composition differed in a species-specific manner with apparent within-species variation (Fig. 1).

### Differences and similarities within and between coral species and seawater

In order to identify differences in the bacterial community composition between different deep-sea corals, sequences were subsampled and clustered into OTUs (Operational Taxonomic Units) (Table 1). Species richness, i.e. the number of different bacterial species present in each coral, was estimated using the Chao1 index^26^. Bacterial species richness estimates ranged from 176 to 1050 taxa for *Dendrophyllia sp*., from 451 to 899 for *E. fistula*, from 442 to 675 for *R. typus*, and from 482 to 599 for seawater. This indicates that species richness in corals was similar to that of the surrounding seawater. Simpson Evenness^27^ ranged from 0.069 to 0.126 for *Dendrophyllia sp*., from 0.010 to 0.079 for *E. fistula*, from 0.020 to 0.031 for *R. typus*, and from 0.019 to 0.045 for sea water. Species diversity (i.e. Inverse Simpson Index^27^) ranged between 11.9 and 85.6 for *Dendrophyllia* sp., 3.2 and 46.7 for *E. fistula*, 2.7 and 11.9 for *R. typus*, and 4.6 and 16.0 for sea water samples. The wide range of bacterial richness and diversity across coral species and in particular for *Dendrophyllia* sp. and *E. fistula* argues for a considerable genotype dependence on community composition^19^.

In all coral species, comparably few OTUs were abundant and made up the majority of all sequences, whereas the majority of OTUs were rare (Supplementary Dataset S1). For instance, for *Dendrophyllia sp.* we identified 1,050 distinct OTUs, with only 13 OTUs each comprising >1% and 1,037 OTUs comprising <1% of all sequences. The most abundant bacterial taxa, OTU0004 (*Pseudomonas veronii*) represented 12% of all sequences. In *E. fistula*, we identified a total of 1,246 OTUs, with 17 OTUs each constituting >1% and 1,229 OTUs constituting <1% of all sequence counts. The most abundant bacterial taxa, OTU0001 (unknown Gammaproteobacterium) comprised 21% of all sequences. Across all *R. typus* samples, we found 842 distinct OTUs, with 13 OTUs constituting >1% and 829 OTUs with <1% of sequence counts. For this species, OTU00003 (unknown Proteobacterium) comprised 17% of all sequences and constituted the most abundant bacterium. Seawater samples contained a total of 557 distinct OTUs, of which 14 contributed >1% and 543 <1% of all sequences. OTU0002 (*Alteromonas* sp.) comprised 24% of all sequences.

**Figure 2:**
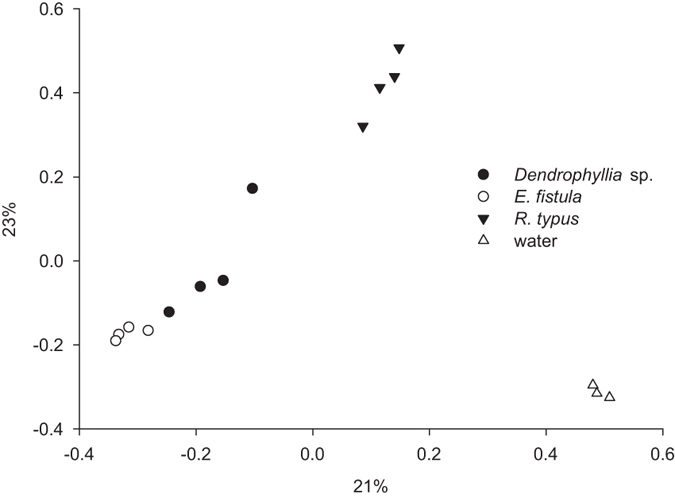
Clustering of deep-sea coral and seawater samples based on bacterial abundances. Ordination is based on a Bray-Curtis dissimilarity matrix and Pearson correlation in a principal coordinate analysis (PCoA) plot (R^2^ = 0.63). Percentages represent the amount variance explained by each dimension.

To further explore differences in bacterial community composition across samples, we plotted a principal coordinate analysis (PCoA) based on a Bray-Curtis dissimilarity matrix (Fig. 2). This showed that sea water samples clustered tightly together and were clearly separated from all coral samples, and coral samples were generally clustering according to species. This was substantiated by AMOVA analysis, which showed that coral were significantly different from seawater (*P*_AMOVA  _=0.002). To further test whether differences between corals exist, we excluded seawater and performed a PERMANOVA across corals. We identified significant differences between coral species (*P*_PERMANOVA_ = 0.0019, Pseudo-F = 3.3554, 4,707 unique permutations) and subsequent pair-wise tests showed all coral species differed significantly from each other (Table S1).

### Core microbiomes

**Figure 3:**
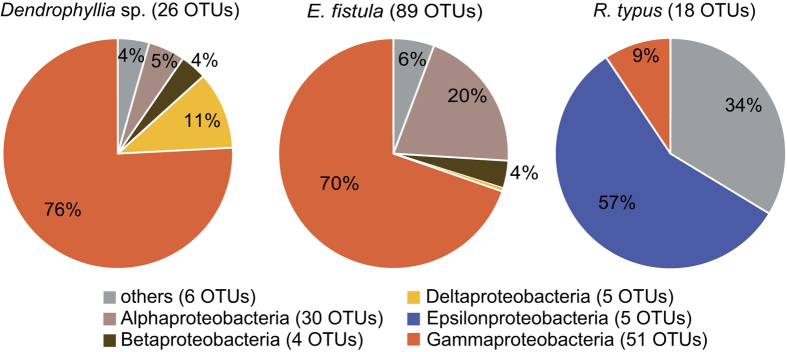
Core microbiomes of three deep-sea coral species from the Red Sea at the bacterial taxonomic class level. Core microbiome members are comprised of bacterial taxa present in 100% of all samples for the species under investigation. Numbers of distinct OTUs in color legend denote bacterial diversity on the class level across coral microbiomes.

Based on the statistically significant differences between coral species, we next assessed differences in absence/presence of individual bacterial taxa across coral species. To do this, we determined coral species-specific core microbiomes. We considered all those OTUs that were ubiquitously present in each species, indicating putative functional importance. Only few bacteria were core microbiome members, in contrast to the large suite of OTUs associated with each species. We identified 26 bacterial taxa in the core microbiome of *Dendrophyllia* sp., 89 in that of *E. fistula*, and 18 in *R. typus*. Of note, all core microbiomes contained rare and abundant bacteria, comprising average abundances between 2 and 977 sequence counts (Supplementary Dataset S1, Supplementary Dataset S2). Core microbiomes largely encompassed bacterial taxa that were unique to a given coral species and only few members were shared between species. For instance, the core microbiome of *Dendrophyllia* sp. harbored 9 exclusive OTUs, *E. fistul*a featured 20 unique bacterial taxa, and 3 bacteria were exclusively found in the microbiome of *R. typus* (Supplementary Dataset S2). In comparison to the core microbiomes of *Dendrophylia* sp. and *E. fistula* that were dominated by Gammaproteobacteria, *R. typus* was dominated by Epsilonproteobacteria (Supplementary Dataset S1, Supplementary Dataset S2, Fig. 3).

## Discussion

To date, only few studies describe microbial communities associated with deep-sea scleractinian corals, all of which have been conducted on *L. pertusa* and *M. oculata* in Norway, the northern Gulf of Mexico, the Mediterranean, and off the coast of Ireland^5^,^16^,^18^,^19^,^20^,^21^,^23^,^24^. Hence, there is a paucity of studies from other species and other environments. The available studies show that coral microbiomes are distinct from the surrounding environment^5^,^16^,^19^,^20^,^21^, differ between different species, sites, and sampling times, and even between individual polys of the same colony^5^,^7^,^18^,^19^,^21^,^23^. Here, we assessed microbial community composition of the deep-sea scleractinian corals *Dendrophyllia sp., E. fistula*, and *R. typus* from the Red Sea to extend the catalog of available coral species and provide insight into putative indications of functional adaption to this unique and warm environment.

In order to characterize differences in the microbiomes from the three coral species *Dendrophyllia* sp., *E. fistula*, and *R. typus*, we assessed alpha and beta diversity indices and identified differentially abundant bacterial taxa. Microbiomes differed significantly between species as reflected by the presence/absence of bacterial taxa as well as by pronounced differences in the abundance of shared OTUs. Interestingly, only two bacterial taxa were consistently present across all replicates and coral species, i.e. *Pseudomonas veronii* (OTU0004) and *Photobacterium angustum* (OTU007), suggesting (1) coral-specific microbiomes and (2) functional importance of these taxa across coral host species. *Pseudomonas veronii* was previously found in shallow-water corals from the Red Sea and was linked to high salinity environmental conditions^28^. Interestingly, several species from the same bacterial genus were identified in *L. pertusa* collected in Norwegian waters^24^. *Photobacterium angustum* (previously named *Vibrio angustum/fischeri*) is described as an endosymbiont in squid, and bacterial species from this genus are shown to inhibit virulence gene expression^29^,^30^. Besides these similarities, the core microbiomes of all three coral species differed markedly. Importantly, core microbiome members are suggested to represent ‘true’ bacterial symbionts given their consistent association with coral host taxa^8^. Only a small proportion of bacterial associates for any coral species were core microbiome members, e.g. 2.5% of all OTUs in *Dendrophyllia* sp., 7.1% in *E. fistula*, and 2.0% in *R. typus*. Nevertheless, these members constituted a substantial portion of all sequences: 51% of all sequences in *Dendrophyllia* sp. were assigned to microbiome members, 73% in *E. fistula*, and 29% in *R. typus*. Unfortunately, a comparison to other deep-sea coral studies remains difficult due to differences in methodology, i.e. cloning-independent (this study) vs. cloning-dependent approaches^5^,^16^,^18^. To our knowledge, there are only two other studies using next-generation sequencing to describe spatial and temporal variable microbiomes of *M. oculata* and *L. pertusa*^23^,^25^. These studies showed that *M. oculata* and *L. pertusa* were dominated by Gammaproteobacteria, Alphaproteobacteria, and Bacteroidetes, and also, that Archaea (Thaumarchaeota marine group I (MGI)) were associated with mucus from *L. pertusa*. Similarly, all species in our study contained Gammaproteobacteria and Alphaproteobacteria, but Bacteroidetes were absent. In the current study, we did not assess archaeal association due to the choice of 16S rRNA gene primers (see Methods). Microbes associated with corals in this study also seemed remarkably consistent for a given coral species in comparison to a previous study that found pronounced differences between colonies^23^. This may be due to a lack of temporal replication, but also, might represent the result from more stable environmental conditions in the deep Red Sea. Conversely, sampling sites in the northwestern Mediterranean Sea are exposed to seasonal changes in nutrient availability and physico-chemical parameters (e.g. temperature)^23^.

Coral habitats from the Red Sea present a unique combination of environmental settings in which coral occur, in particular in regard to depth distribution and dissolved oxygen (DO)^9^,^11^,^31^. Compared to other deep-sea habitats, corals from the Red Sea are exposed to substantially warmer temperatures (>20 °C), considerably lower DO levels (<2 mg L^−1^), and highly oligotrophic waters^10^,^11^. In particular, high water temperatures in combination with low nutrient and oxygen levels are challenging for corals, prompting the suggestion of specific adaptations to the environment of the Red Sea^11^,^31^,^32^. Interestingly, however, deep-sea coral species found in the Red Sea (e.g., *E. fistula* and *R. typus*) are not endemic and exist in other deep-sea environments, such as the IndoPacific and Oceania^33^. It is unclear at present how these corals adapt to the prevailing environmental conditions of the Red Sea. For *E. fistula*, substantial physiological plasticity has recently been demonstrated^34^, and it would be intriguing to see whether this plasticity could be linked to flexibility in microbial community structure.

Along this line of thought, microbiomes in shallow water corals are suggested to play an important role for coral health^35^,^36^,^37^,^38^. This is presumably even more so for azooxanthellate corals^5^,^6^,^22^. In particular, it has been suggested that deep-sea coral associated bacteria may enhance nutrient cycling, serve as nutrition, enable cellulose catabolism, sulphur cycling, and anoxic fermentation^5^. Most recently, Middelburg *et al*.^6^ showed that microbes provide symbiotic nitrogen fixation, chemoautotrophy, and a high capability for nutrient recycling in *L. pertusa*, but these pathways are not yet linked to specific microbial groups.

Anoxic conditions in micro-niches have been discussed in deep-sea corals^5^ and the low oxygen levels present in the deep Red Sea may promote association with anaerobic bacteria. We found anaerobic bacteria from the genera Propionibacterium and Planctomyces associated with corals in this study, although their abundances were comparably low (Supplementary Dataset S1). Conversely, we identified bacteria of the families Rhodobacteraceae and Rhodospirillaceae in high abundance. Both families include members connected to anaerobic conditions^5^. Lastly, bacteria from the genus *Vibrio* (present at comparably low abundances in our samples) are capable of nitrogen fixation, in particular under low oxygen or anoxic conditions^39^,^40^.

Nitrogen fixation and carbon acquisition are prerequisites for corals to survive in low-nutrient environments, such as the Red Sea. In this context, we identified Rhodobacteraceae that are capable of metabolizing nitrogen and carbon from chitin in prey crustaceans. In addition, this bacterial family includes denitrifiers supporting efficient nitrogen cycling in corals^5^. The abundance of Rhodobacteraceae varied strongly within and between species (152 ± 128 for *Dendrophyllia* sp., 38 ± 31 for *E. fistula*, and 512 ± 684 in *R. typus*), which might align with availability of heterotrophic feeding sources that potentially subsidize for bacterial-assisted metabolic complementation.

Members of the family Rhodobacteraceae have further been identified as key players in oil degradation in the marine environment^41^. Interestingly, in our samples, besides Rhodobacteraceae, we identified additional bacteria capable of degrading crude oil compounds. For instance, *Pseudomonas veronii* was present in all samples and commonly at high abundance. *P. veronii* is capable of degrading a variety of organic compounds including alkylphenols^42^ and alkyl methyl ketones^43^, and to absorb heavy metals^44^, which are also found in crude oil^45^,^46^,^47^. We further identified *Methylibium petroleiphilum*^48^, *Oleibacter sp.*^49^, *Acinetobacter venetianus*^50^*, Marinobacter bryozoorum*^41^, *Thalassospira xiamenensis*^51^, and *Aestuariicella hydrocarbonica* (OTU0009, annotation to NCBI ‘nr’, 99% sequence similarity)^52^, all of which have been described to possess the potential to degrade crude oil (Supplementary Dataset S1). Considering all oil-degraders that could be assigned to the species level, about 30% of all sequences for *Dendrophyllia* sp., 5% for *E. fistula*, and only 0.2% for *R. typus* were on average comprised by these bacteria. Hence, the presence of oil-degraders does not seem to be a general characteristic of deep-sea corals from the Red Sea, although oil degraders have been suggested to play a functional role in (shallow-water) coral holobionts^53^. Crude oil and its derivatives may therefore constitute a carbon source for deep-sea corals (in particular *Dendrophyllia* sp.) in the deep Red Sea^54^, but these assumptions would need to be substantiated by stable isotope data showing incorporation of the hydrocarbon signal into the coral tissue. The presence of anaerobic bacteria, however, seems to be a general hallmark that warrants further study into their contribution to coral functioning in the unique environment of the deep Red Sea.

## Conclusion

In this study we characterized microbial communities associated with three deep-sea corals from the Red Sea. All corals exhibited species-specific bacterial associations and specific bacterial taxa provide support to the notion that bacteria contribute to environmental adaption of their coral hosts. These bacteria include obligate or facultative anaerobic bacteria that may aid the coral to survive in the low oxygen and warm environment of the deep Red Sea. We further identified bacteria associated with degrading crude oil compounds, potentially suggesting the utilization of crude oil and its derivatives as a carbon source for deep-sea corals from the Red Sea. To further understand functional implications of bacterial association, microbial characterization of congenerics from these coral species outside the Red Sea is desirable.

## Methods

### Study site and sample collection

Corals in this study were collected by remotely operated vehicle (ROV) during a central and northern Red Sea expedition (KRSE2013L6) from 11–22 May 2013 on the R/V Aegaeo (operated by the Hellenic Center for Marine Research, Greece). Three coral species, *Dendrophyllia sp., Eguchipsammia fistula*, and *Rhizotrochus typus* were collected each with four biological replicates for a total of 12 coral samples. Upon collection, *E. fistula* and *R. typus* samples were placed into a custom-made two-compartment container and preserved in RNAlater at depth. *Dendrophyllia* sp. samples were collected in a plastic basket and brought back to the surface within 90 min of sampling. For each site, one seawater sample was collected from the vicinity of the collection site via Niskin bottles during additional dives. *Dendrophyllia sp.* was collected between 625 and 630 m depth (N22˚46.167′, E38˚03.102′), *E. fistula* was collected between 314 and 320 m depth (N22˚17.837′, E38˚53.811′), and *R. typus* was collected between 970 and 993 m depth (N27˚44.215′, E35˚08.000′). Samples from each coral species were collected on separate ROV dives to prevent cross contamination. Directly after retrieval, coral samples were rinsed with filtered seawater, crushed on liquid nitrogen and stored in cryotubes at −80 °C until DNA extraction (see below). One liter of each seawater sample was filtered over a 0.22 μm Durapore^®^ filter (Millipore, Billerica, USA), and filters were stored at −80 °C until DNA extraction.

### DNA extraction and 16S rRNA gene amplicon sequencing

DNA from crushed coral was isolated using the AllPrep DNA/RNA Mini kit (Qiagen, Hilden, Germany) following the manufacturer’s protocol. For DNA isolation from seawater samples, 1200 μl of buffer RLT (Qiagen) were used on a quarter of a filter and incubated for 20 minutes on a tabletop rotator (Sb2 rotator, Stuart, Staffordshire, UK). DNA isolation commenced following the manufacturer’s instructions. Sequencing libraries were generated following the Illumina 16S metagenomic sequencing library preparation protocol. 16S rRNA locus was amplified using the primers 784F (5′-TCGTCGGCAGCGTCAGATGTGTATAAGAGACAGAGGATTAGATACCCTGGTA-3′) and 1061R (5′-GTCTCGTGGGCTCGGAGATGTGTATAAGAGACAGCRRCACGAGCTGACGAC-3′) (Illumina overhang adaptor sequences underlined)^55^. These primers were found to amplify bacterial 16S rRNA sequences well across a range of coral species^56^, but are limited in their ability to amplify archaeal 16S rRNA sequences. Hence, archaeal 16S rRNA sequences were removed prior to microbial community composition analysis (see below). Of note, archaea associate with scleractinian corals, but seem to be far less abundant than bacteria^57^,^58^. PCRs were run in triplicate at a volume of 20 μl using the Qiagen multiplex PCR Master Mix with ~50–100 ng DNA for coral and ~1–2 ng DNA for seawater samples, respectively. The final primer concentration was adjusted to 0.25 μM. The PCR conditions were set as follows: 95 °C for 15 minutes, followed by 25 cycles of 95 °C for 30 seconds, 55 °C for 90 seconds and 72 °C for 30 seconds, with a final extension time of 10 minutes at 72 °C. Triplicate PCRs for each sample were pooled and amplification was visualized on the Bioanalyzer (Agilent Technologies, Santa Clara, USA). Samples underwent an index PCR amplification following the Illumina 16S metagenomic sequencing library preparation protocol, combined and run on a 2% ultra-pure agarose gel (Ultrapure Agarose, Life Technologies), and cut out on a non-UV transilluminator (Dark Reader, Clare Chemical Research, Dolores, USA) to remove excess primer. DNA was purified following the Zymoclean DNA large fragment recovery kit (Zymo Research, Irvine, USA) with two elutions at 10 μl. The 16S rRNA gene amplicon libraries were sequenced on one lane on the Illumina MiSeq platform with 25% phiX control at the KAUST sequencing facility. The libraries were sequenced using 2 × 300 bp overlapping paired-end reads.

### Bacterial community analysis

Bacterial community analysis was conducted using mothur (v.1.36.1)^59^. Briefly, sequence reads were trimmed and read pairs were merged into contigs. Contigs longer than 306 bp and those with ambiguously called bases were discarded. Sequences that occurred only once were discarded. The sequences were then pre-clustered, allowing for up to a 2 base pair difference between sequences. Chimeras were removed using UCHIME, as implemented in mothur. Sequences were classified with the Greengenes database^60^ using a 60% bootstrap cutoff, and mitochondria, chloroplasts, archaea, eukaryote, and unknown sequences were removed. All samples were subsampled to 4,600 sequences, corresponding to the lowest number of sequences available for any sample. Bacterial community compositions were visualized using pie charts and a stack column plot based on annotated sequences. Subsampled sequences were clustered into operational taxonomic units (OTUs) with a similarity cutoff of 97%. Alpha diversity indices (Chao1, Simpson Evenness, and Inverse Simpson Index) were calculated using mothur. Beta diversity differences were visualized in a principal coordinate analysis (PCoA) based on a Bray-Curtis dissimilarity matrix and Pearson correlation. Differences between coral and seawater samples were assessed using Analysis of Molecular Variance (AMOVA) in mothur. A one-factorial permutational MANOVA (PERMANOVA) analysis was conducted using PRIMER v6 software (PRIMER–E Ltd, Ivybridge, UK)^61^ on square root transformed OTU abundance counts. The test design was based on Bray-Curtis similarities, partial sum of squares type III, 9,999 permutations of residuals under a reduced model using Monte-Carlo simulations, and followed by pair-wise tests. Core microbiomes were determined following a conservative approach, considering only OTUs that occurred in every sample of a given coral species.

## Declarations

### Ethics approval and consent to participate

Experimental research detailed in this study complies with institutional guidelines following KAUST Institutional Biosafety and BioEthics Committee (IBEC).

## Additional Information

**Accession Codes**: Raw sequence data determined in this study have been deposited on NCBI under BioProject Accession No. PRJNA354830 (https://www.ncbi.nlm.nih.gov/bioproject/PRJNA354830). Core microbiome OTU reference sequences are available under GenBank Accession numbers KY515469 - KY515585.

**How to cite this article:** Röthig, T. *et al*. Microbial community composition of deep-sea corals from the Red Sea provides insight into functional adaption to a unique environment. *Sci. Rep.* **7**, 44714; doi: 10.1038/srep44714 (2017).

**Publisher's note:** Springer Nature remains neutral with regard to jurisdictional claims in published maps and institutional affiliations.

## Supplementary Material

Supplementary Information

Supplementary Dataset S1

Supplementary Dataset S2

## Figures and Tables

**Table 1:** Summary statistics of 16S rRNA-based bacterial community composition of deep-sea coral and seawater samples.

Sample Name	No. of Sequences	No. of OTUs*	Chao 1*	Simpson Evenness*	Inverse Simpson Index*
*Dendrophyllia* sp. 1	20,984	682	1050	0.126	85.6
*Dendrophyllia* sp. 2	4,607	173	176	0.069	11.9
*Dendrophyllia* sp. 3	28,345	230	295	0.070	16.1
*Dendrophyllia* sp. 4	68,562	229	262	0.083	19.1
AVG	30,625	329	446	0.087	33.2
SD	27,168	237	406	0.027	35.1
*E.fistula* 1	58,909	657	899	0.067	44.3
*E.fistula* 2	21,485	589	839	0.079	46.7
*E.fistula* 3	10,830	318	451	0.019	6.1
*E.fistula* 4	33,871	337	468	0.010	3.2
AVG	31,274	475	664	0.044	25.1
SD	20,690	173	238	0.035	23.6
*R. typus* 1	12,664	452	675	0.026	11.9
*R. typus* 2	39,931	202	442	0.031	6.3
*R. typus* 3	63,947	241	586	0.020	4.7
*R. typus* 4	56,370	191	451	0.014	2.7
AVG	43,228	272	538	0.023	6.4
SD	22,708	122	112	0.007	3.9
Seawater1	45,931	265	506	0.024	6.4
Seawater2	32,959	244	482	0.019	4.6
Seawater3	54,693	358	599	0.045	16.0
AVG	44,528	289	529	0.029	9.0
SD	10,935	61	62	0.014	6.1

^*^Based on subsampled sequences (n = 4,600).

AVG = average; SD = standard deviation.
