# Supplementary material for: Microbial community composition of deep-sea corals from the Red Sea provides insight into functional adaption to a unique environment
Source: Sci Rep. 2017 Mar 17;7:44714. doi: 10.1038/srep44714 (PMC5356181; doi:10.1038/srep44714)
Supplement: Supplementary Information [file srep44714-s1.pdf]

Supplementary Information

Microbial community composition of deep-sea corals from the Red Sea provides insight into functional adaption to a unique environment

Till Röthig[1, +], Lauren K. Yum[1, +], Stephan G. Kremb[1], Anna Roik[1], Christian R Voolstra[1,*]

[1]Red Sea Research Center, Division of Biological and Environmental Science and Engineering (BESE), King Abdullah University of Science and Technology (KAUST), Thuwal 23955-6900, Saudi Arabia

[+]Authors contributed equally

[*]Corresponding author:

Email: christian.voolstra@kaust.edu.sa

Phone: +966 54 470 0087

Fax: +966 21 8082377

Short Tile: Microbial communities of deep-sea corals from the Red Sea

Keywords: Red Sea, deep-sea coral, microbial community profiling, 16S rRNA gene, climate change

Table S1. Statistical overview of pair-wise PERMANOVA results. PERMANOVA analysis based on a Bray-Curtis resemblance matrix of square root transformed OTU abundances. Test design with sums of square type III (partial) and 9,999 permutations.

Groups	t	*P*(perm)	Unique Permutations	P(MC)
Dendrophyllia sp., *E. fistula*	1.7469	0.0311	35	0.0256
Dendrophyllia sp., *R. typus*	1.6985	0.0297	35	0.0368
E. fistula, *R. typus*	2.0534	0.0299	35	0.0085

Supplementary Dataset S1. OTU abundance over all samples for *Dendrophyllia* sp., *Eguchipsammia fistula*, and *Rhizotrochus typus* including annotation, and reference OTU sequence.

Supplementary Dataset S2. Overview of core microbiome members of *Dendrophyllia* sp., *Eguchipsammia fistula*, and *Rhizotrochus typus*. Core microbiome members of each respective coral species are indicated in **bold**. Numbers denote average OTU abundance (± SE) for each coral species and the respective annotation for each OTU (with bootstrap support).
